# Supplementary material for: End Sequence Analysis Toolkit (ESAT) expands the extractable information from single-cell RNA-seq data
Source: Genome Res. 2016 Oct;26(10):1397–410. doi: 10.1101/gr.207902.116 (PMC5052061; doi:10.1101/gr.207902.116)
Supplement: Supplemental Material [file supp_gr.207902.116_Supp_Materials.tar.gz › ESAT-esat_v0.1/doc/html/classumms_1_1core_1_1coordinatesystem_1_1_transcriptome_space_1_1_gene_tree.html]

ESAT: umms.core.coordinatesystem.TranscriptomeSpace.GeneTree Class Reference


ESAT


- Main Page
- Related Pages
- Packages
- Classes
- Files

- Class List
- Class Index
- Class Hierarchy
- Class Members

All Classes Namespaces Files Functions Variables Enumerator Pages

- umms
- core
- coordinatesystem
- TranscriptomeSpace
- GeneTree

Public Member Functions |
List of all members

umms.core.coordinatesystem.TranscriptomeSpace.GeneTree Class Reference

Collaboration diagram for umms.core.coordinatesystem.TranscriptomeSpace.GeneTree:

[legend]

|  |  |
| --- | --- |
| Public Member Functions | |
| Collection< GeneWindow > | getRegion (Annotation region) |
|  | |
| Collection< GeneWindow > | getRegion (String chr) |
|  | |
| Collection< GeneWindow > | getRegion () |
|  | |
| long | getLength () |
|  | |
| long | getLength (String chr) |
|  | |
| Annotation | getRandomAnnotation () |
|  | |

## Member Function Documentation

|  |  |  |  |  |
| --- | --- | --- | --- | --- |
| long umms.core.coordinatesystem.TranscriptomeSpace.GeneTree.getLength | ( |  | ) |  |

Here is the call graph for this function:

|  |  |  |  |  |  |
| --- | --- | --- | --- | --- | --- |
| long umms.core.coordinatesystem.TranscriptomeSpace.GeneTree.getLength | ( | String | *chr* | ) |  |

Here is the call graph for this function:

|  |  |  |  |  |
| --- | --- | --- | --- | --- |
| Annotation umms.core.coordinatesystem.TranscriptomeSpace.GeneTree.getRandomAnnotation | ( |  | ) |  |

|  |  |  |  |  |  |
| --- | --- | --- | --- | --- | --- |
| Collection<GeneWindow> umms.core.coordinatesystem.TranscriptomeSpace.GeneTree.getRegion | ( | Annotation | *region* | ) |  |

Returns the trimmed region contained in this interval

Parameters
:   |  |  |
    | --- | --- |
    | chr |  |
    | start |  |
    | end |  |

Returns

Here is the call graph for this function:

|  |  |  |  |  |  |
| --- | --- | --- | --- | --- | --- |
| Collection<GeneWindow> umms.core.coordinatesystem.TranscriptomeSpace.GeneTree.getRegion | ( | String | *chr* | ) |  |

Returns all genes that have chr

Parameters
:   |  |  |
    | --- | --- |
    | chr |  |

Returns

|  |  |  |  |  |
| --- | --- | --- | --- | --- |
| Collection<GeneWindow> umms.core.coordinatesystem.TranscriptomeSpace.GeneTree.getRegion | ( |  | ) |  |

Returns all genes

Returns

---

The documentation for this class was generated from the following file:

- java/umms/core/coordinatesystem/TranscriptomeSpace.java


---

Generated on Fri Aug 15 2014 15:11:10 for ESAT by  

 1.8.7
